# A Study on the Design of Fog Computing Architecture Using Sensor Networks

**DOI:** 10.3390/s18113633

**Published:** 2018-10-26

**Authors:** Hyun-Jong Cha, Ho-Kyung Yang, You-Jin Song

**Affiliations:** 1 Division of Information Technology Education, Sunmoon University, Asan-si 31460, Korea; chj826@kw.ac.kr (H.-J.C.); porori0421@naver.com (H.-K.Y.); 2 Department of Management, Dongguk University, Gyeongju-si 38066, Korea

**Keywords:** IoT, fog computing, sensing data, architecture

## Abstract

It is expected that the number of devices connecting to the Internet-of-Things (IoT) will increase geometrically in the future, with improvement of their functions. Such devices may create a huge amount of data to be processed in a limited time. Under the IoT environment, data management should play the role of an intermediate level between objects and devices that generate data and applications that access to the data for analysis and the provision of services. IoT interactively connects all communication devices and allows global access to the data generated by a device. Fog computing manages data and computation at the edge of the network near an end user and provides new types of applications and services, with low latency, high frequency bandwidth and geographical distribution. In this paper, we propose a fog computing architecture for efficiently and reliably delivering IoT data to the corresponding IoT applications while ensuring time sensitivity. Based on fog computing, the proposed architecture provides efficient power management in IoT device communication between sensors and secure management of data to be decrypted based on user attributes. The functional effectiveness and the safe data management of the method proposed are compared through experiments.

## 1. Introduction

With the development of the Internet of Things (IoT), a device could recognize the environment and conduct a certain function by itself. Therefore, roles of a computing model that controls IoT devices are paramount. IoT devices mainly consist of sensors. Previously, cloud computing based on a sensor network was used to manage sensor data [1,2]. Transferring and processing huge amount of data through cloud computing caused several issues such as delayed service response time. In addition, the number of application area of a sensor network is expanding and the demand on real-time processing and transferring of information to control a device of IoT is also increasing [3].

A new type of computing model, fog computing, is proposed as the amount of data generated by a sensor is increasing and a routine to process the data is complicate. Fog computing is a model to manage data, conducting near field communication with a sensor. As a device in fog computing uses near field communication, its response speed is faster than cloud computing and the number of sensors allocated to a device is smaller than in cloud computing. Therefore, more and faster tasks are possible, compared to cloud computing. Considering the fact that it requires data aggregation to reduce waste of energy caused by duplicate delivery of information between near devices in a sensor network, clustering-based hierarchical management has many advantages [4,5,6].

**Figure 1:**
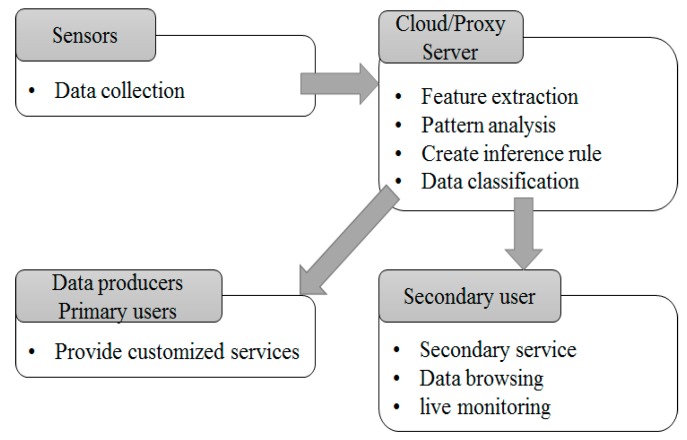
Data flow according to access authority.

In addition, fog computing services that have been developed so far consist of data creation, processing and transfer, as shown in Figure 1. In other words, a cloud server or a proxy server extracts features according to a type of data once data is created at a sensor. Then, a server creates rules of pattern analysis and inference to save or process data. Data processed through the process is provided to a data owner or a user in customized service. In such systems, data for a service user was saved in a plain text type and transferred to a monitoring server for future access and use of a user. Data sharing and utilization are inevitable functions. Data generating at a sensor can be fatal information to an individual user, therefore, a delegation function to allocate the authority of data access to a legitimate user and revocation function to remove the authority of data access from the user are required.

This paper proposes an effective solution to resolve an unnecessary power consumption issue in communication between sensors under a fog computing environment. In addition, a fog computing architecture for safe management of data generating at each sensor is proposed. The proposed architecture is based on IoT reference model proposed by CISCO. At the application level, a data security method through user attribute-based encryption and decryption and delegation and revocation of authority is proposed. At a lower level, unnecessary communication is controlled by expecting usage and frequency of communication between sensors.

This paper consists of the following sections. The second section reviews the Internet of Things, the system environment proposed in Section 2, attribute-based encryption for fog computing and secure data management and a sensor network that is the base of communication between devices. Section 3 proposes a hierarchical management method that expects power consumption and frequency of use of a sensor to improve overall architecture and communication between devices as a description of the system proposed. In addition, a method to delegate or remove authority in attribute-based encryption for secure management. Section 4 evaluates performance of a proposed method. Finally, Section 5 describes the conclusions of this study and possible topics for future study.

## 2. Related Research

### 2.1. Internet of Things

**Figure 2:**
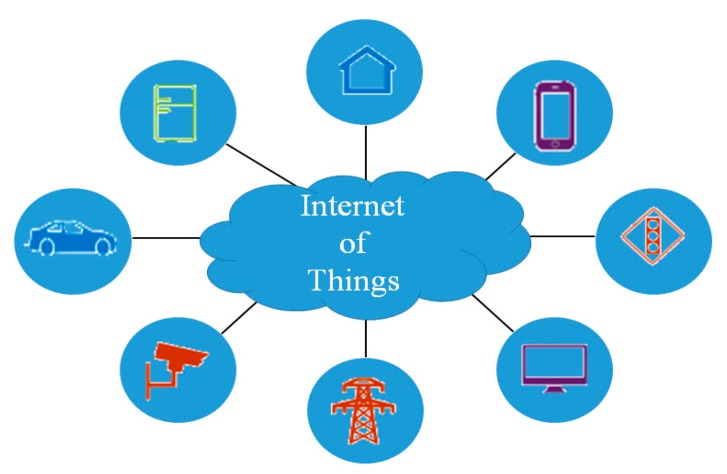
Concept of the Internet of Things.

Internet of Things (IoT) is the intelligent environment that allows mutual communication between users and things and things and things by connecting all things through wired or wireless networks, based on Internet Communication Technology (ICT). In other words, the IoT makes things that are working under different operation systems, network environment and hardware environment interoperable through the Internet. Figure 2 shows the concept of IoT.

Gartner, the U.S. market research institution, nominated the Internet of Things at the top of its list of best popular technologies [7]. The IoT can be classified into three major areas: device area, network (wired and wireless) area and service interface (platform and application) area. The device area transfers data collected or drawn from a certain object to other objects, using the built-in communication functions of an object. The network area is a wired or a wireless path for data transmitted between users and things or things and things. The service interface area processes data to create information and control and manage the various devices.

CISCO defines standard terms for each level and describes functions of each level and interaction between levels, introducing the reference model of IoT [8]. The reference model consists of 7 levels. Each level does not limit the scope of a corresponding component or restricts a region. For example, Table 1 indicates IoT reference model and associated levels proposed by CISCO. The data direction is defined as bi-directional flow. Control information in the control pattern flows from the top (Level 7) to the bottom (Level 1). Information flow in the monitoring pattern is opposite. Information flow in most systems is bi-directional.

### 2.2. Fog Computing

Edge computing is a concept that contrasts with cloud computing. Cloud computing is a way to communicate directly with a central data center, whereas edge computing communicates primarily with the so-called “edge data center,” which is located near the device, and leaves secondary work to the central cloud. In other words, edge computing is a computing topology concept.

Fog computing was first proposed by CISCO in January 2014. Antunes, senior director of corporate strategy innovation at CISCO, has stated that edge computing is a component or subset of fog computing. He said: “fog computing seems to be a way to handle where data is generated from where it is stored. Edge computing is simply to be processed near the point where the data was generated. Fog computing includes not only its edge processing but also the network connections necessary to import that data from the edge to the endpoint“. Fog computing refers to the network connections between edge devices and the cloud. On the other hand, edge computing refers more specifically to computing processes performed near edge devices. Therefore, fog computing includes edge computing, as well as networks that are required to send processed data to the final destination. In other words, it is a standard that defines how edge computing should work. It creates a rapid control loop, using a fog computing model, because data is processed at the device [9].

**Figure 3:**
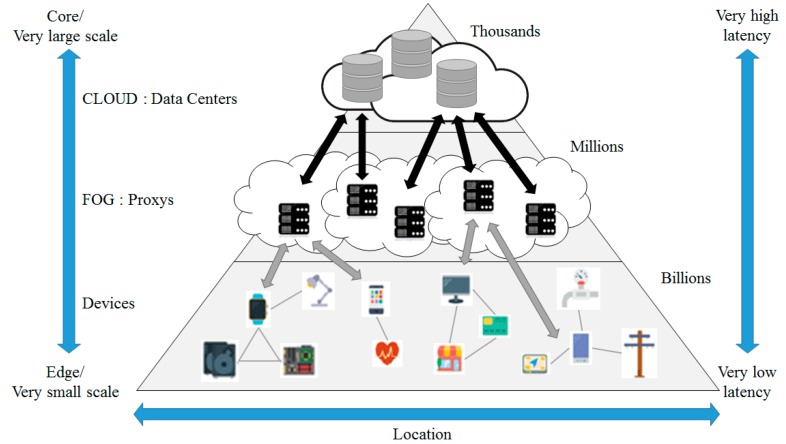
The Structure of Fog Computing.

The structure of fog computing is shown in Figure 3.

Fog computing [9,10,11] is a virtual platform that provides processing, saving and networking service between a device and a cloud computing data center. However, it is not exclusively located at the edge of network. Processing, saving and networking resources are building blocks of cloud and fog.

The cloud layer, which is the core of fog computing, performs data virtualization, analysis, machine learning, and updates rules and patterns in the fog layer’s proxies. The proxy server serves as a simpler cloud server. A concentrated data storage provides creditability and easy access to data by computing resources in a cloud. A data storage that is located at the center of the fog computing structure can be accessed by both device layer and fog layer [12]. The fog computing structure provides some advantages as follows:

- Reduced network load: In the fog computing structure, the amount of data flowing into a network is reduced because computation is conducted at a network edge near IoT devices.
- Mobility support as a default function: The Mobility according to reliability is a fundamental requirement to many IoT applications. The device resources like smart phones and laptops may provide physical or virtual mobility to support a mobile IoT application.
- Context awareness: In the fog computing structure, resources provide context awareness relating to data created by a sensor. The device resources play roles in combining data at a sensor, using position or application context.
- No single defective point: As calculation is completed in a distributed way in fog computing, the model does not have a single defective point. Several snapshots of an application can be allocated at a cloud to improve reliability.

### 2.3. Sensor Network

**Figure 4:**
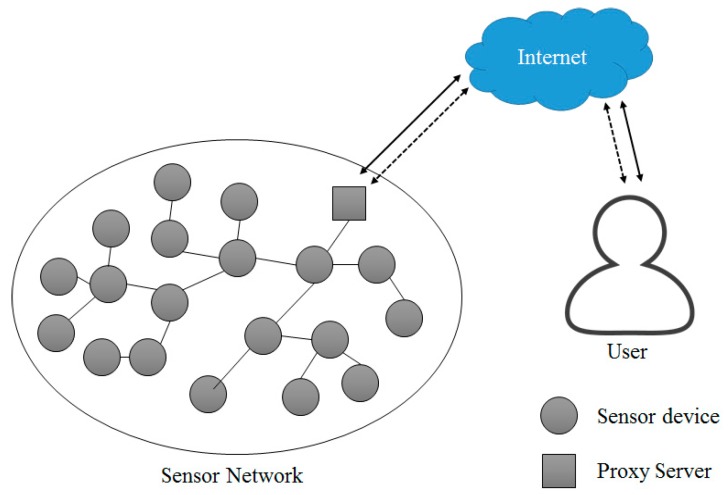
Sensor Network Configuration in the IoT environment.

Due to the development and commercialization of wireless communication technology, sensor network technology has attracted great attention. A sensor network is the environment to connect hundreds of devices. Therefore, wireless sensor network-based products which allow configuring a sensor network environment, have a large market [13]. Figure 4 shows structure of a sensor network in the IoT environment.

Under the sensor network environment, devices are distributed at various areas and form a network, making it impossible to collect devices, replace hardware and replace or charge a battery. Under a sensor network environment, research and development of network and protocol technology have been conducted to improve energy efficiency, considering the hardware limitations of devices. 

A variety of conditions including hardware limitations, network topology, the communication environment and power consumption must be considered to construct the sensor network environment. In general, sensor devices in a sensor network adopting a cluster-based protocol transmit measured data to the m-proxy, which is a small unit proxy server of fog computing. The m-proxy server is a device that collects data from the network in the cluster structure and transfers it to the proxy server in fog computing. The proxy server is a system that collects sensing information sensed by a sensor device, or links event data to the outside of the sensor network and manages the related sensor network. Due to the development of technology, recent sensor device are becoming low cost, low power, and miniaturized. However, they have also some technical difficulties such as replacement of hardware and replacement or charge of battery. Studies on the development of technology to overcome such shortcomings are currently undergoing.

Considering the fact that it requires data aggregation to reduce waste of energy caused by duplicate delivery of information between near devices in a sensor network, clustering-based hierarchical management has many advantages. In other words, fog computing creates a local cluster, transfers similar information about events happening at a near area to the m-proxy server, realizes energy effective routing by allowing the m-proxy server to perform data aggregation, and prevents flooding of ineffective inquiries by transferring requested inquiries through the m-proxy server.

**Figure 5:**
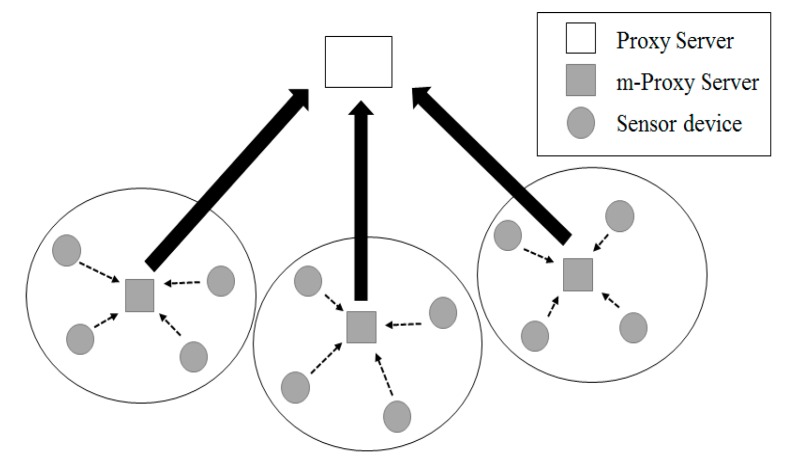
Clustering of Sensor Networks in an IoT environment.

A sensor network in IoT environment adopting clustering algorithm is divided into smaller areas called a cluster, as shown in Figure 5. A cluster has an m-proxy server. An m-proxy server collects data from cluster members and forwards the collected data to a proxy server.

One of the most layered routing protocols used in sensor networks is the Low Energy Adaptive Clustering Hierarchy (LEACH) protocol. LEACH is a method to periodically replace m-proxy servers with the highest energy consumption based on probabilities. It prolongs the lifetime of the sensor network compared to existing routing protocols. However, a certain number of m-proxy servers are not selected for each round by a probabilistic m-proxy server determination method and the m-proxy servers can be concentrated in a specific area in the sensing field. To solve this problem, a LEACH-Centralized (LEACH-C) routing protocol that complements LEACH has been proposed, but LEACH-C also requires additional energy consumption for all devices to communicate with the proxy server in every round, so additional overhead occurs. In addition, the data transmission process between the proxy server and the sensor devices is the same as the clustering-based routing technique. However, in this technique, in selecting the m-proxy server differently from LEACH, the m-proxy server is selected according to the position information of the devices and the energy holding amount. Since this method knows the location information of the devices in advance, it is possible to appropriately distribute the m-proxy servers, thereby making it possible to construct a more robust network. However, since each device frequently communicates with the proxy server for its current remaining energy level transmission, so the communication cost increases.

### 2.4. Attribute-Based Encryption

Attribute-based encryption is a type of encryption method that allows only users who have sufficient attributes about encrypted data to decrypt the data. Attribute-based encryption starts from configuring identity values of identity-based encryption to a set of pre-determined attributes. The attribute-based method proposed by Sahai, et al. in an early stage, used a threshold technique. A ciphertext contains Set S consisting of attributes owned by an encryptor. A user who wants to decode a ciphertext compares Set S’ (a group of his/her own attributes) with Set S. If a certain number (k) of attributes is matched, the cipher text can be decrypted.

After that, there have been many studies on diversified message encryption techniques, beyond the limitations of the threshold technique, which is a simple comparison of attribute values. As a result, two attribute-based encryption methods were proposed: key policy attribute-based encryption and ciphertext policy attribute-based encryption. 

The ciphertext-policy attribute-based encryption proposed by Bethencourt et. al solves the issue of key-policy attribute-based encryption in which an encryptor does not have an authority to access a ciphertext as a user [14]. A ciphertext created by ciphertext-policy attribute-based encryption contains access tree structure and encrypted data which are required to decrypt a ciphertext. A private key to be used consists of keys issued by a key issuer against attributes of a user. If an attribute value owned by a user is matched with an access tree structure included in a ciphertext, the ciphertext can be decoded.

**Figure 6:**
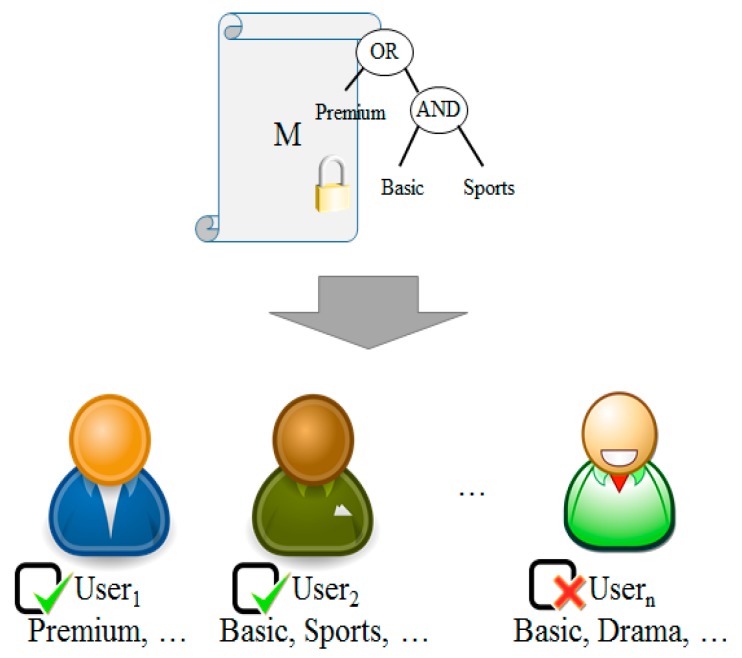
Ciphertext-Policy Attribute-Based Encryption.

The process of ciphertext-policy attribute-based encryption is illustrated in Figure 6. Each user has an attribute such as ‘Premium’, ‘Basic’ or ‘Drama’. In a message, an access tree of ‘Premium’ ∨ (‘Basic’ ∧ ‘Sports’) is encrypted. User 2 also can decrypt the message because the access tree is satisfied. However, User M cannot decrypt the message because the access tree is not satisfied.

The existing Ciphertext-Policy Attributed-Based Encryption (CP-ABE) method does not practically deal with revocation and delegation. It conducts flexible attribute delegation and revocation as an extended type of attribute-based encryption. Ciphertext-Policy Attributed-Based Threshold Decryption (CP-ABTD) has three characteristics: first, a delegator who owns a private key about an attribute group can assign his/her authority to a delegatee. Second, a delegator can decide delegation of his/her authority to a delegatee. Third, the proposed method allows revocation of attributes [15].

Most of the methods proposed in attribute-based encryption use a list of people who revoke attributes and NOT operations in Key-Policy Attribute-Based Encryption (KP-ABE). An example of an attribute revocation method is to set the expiry date on attributes.

Bethencourt briefly mentioned how to revoke an attribute value, proposing CP-ABE [4]. The method to revoke attributes is to attach an expiry date to the private key and an attribute value of a ciphertext. The expiry date of an attribute value included in the private key is compared with the time value of a ciphertext. If the expiry date is passed, a user is not allowed to use an attribute value any more. However, this method does not propose how to renew expired attribute values. Therefore, the expiry date is passed, an attribute must be re-issued. In addition, this method does not consider how to revoke an attribute value before the expiry date.

To propose how to revoke an attribute value in KP-ABE, Ostrovsky allocates the revocation list to a ciphertext [16]. In addition, he creates a d-order equation, assuming that all ciphertext has ‘d’ (number) attribute values. A user who is not included in the revocation list uses his/her ID as an attribute value and calculates the private value through interpolation with a result from ‘d+1’ polynomial equations. If a user is included in the revocation list, he/she could have only ‘d’ results and could not have a private value. As this method fixes the number of attribute value to ‘d’, effectiveness and efficiency are low. In addition, the revocation list should continue to be maintained, rather renewing attribute values owned by a revoker.

Attrapadung proposed Direct Revocation and Indirect Revocation methods for CP-ABE and KP-ABE. Direct revocation directly revokes a key using the service revocation list [17]. However, this method should keep maintaining the list. Indirect revocation has an authentication server. A user who is not on the revocation list revokes an attribute value by communication with the authentication server [18,19]. This method is not required to maintain the revocation list but may create bottlenecks because of the necessary continual communication with the authentication server.

## 3. Proposed System

### 3.1. Platform Design

**Figure 7:**
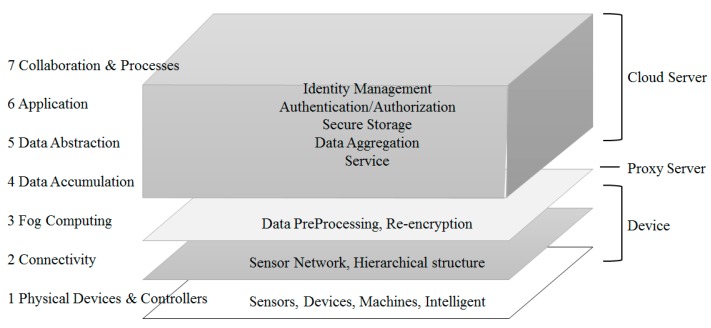
Role for Each Layer of the Proposed System.

As shown in Figure 7, the platform divided into the role-based hierarchy of the proposed object Internet consists of the cloud, the proxy server, and the device. A proxy server has a server to analyze data and a storage to save data. A proxy server conducts re-encryption. A proxy server monitors device information and controls devices according to the information analyzed.

**Figure 8:**
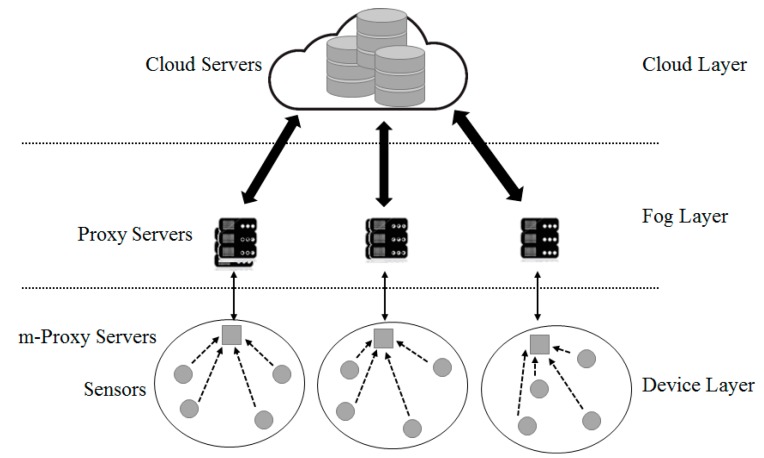
Proposed System Architecture.

Connection types of devices differ according to the specifications of the connected device. Unlike a high performance device, as a low performance device is configured with simple sensors and an actuator only, it is difficult to communicate with a proxy server. To deal with this issue, select a high performance device as a small segment of a proxy server and make it play an intermediate role for a proxy server. The system proposed consists of three layers, as illustrated in Figure 8. Each layer has a cloud, a fog, and a device layer.

The top layer is the cloud computing layer that receives information from a proxy server. It also plays the role of a storage device to save all data inside the cloud. The saved data can be used for other purposes, such as data mining and management. The fog layer is a highly virtualized platform that provides a number of services such as calculation, saving and networking services at the network edge near an IoT device. It makes cloud service available near IoT devices or mobile users.

The device layer includes an m-proxy server with a limited amount of resources, which is installed at a store, a cafe or a public facility such as an intersection within one or two hops from a sensor or an IoT device to collect information. As calculation is conducted at a network edge near an IoT device, it reduces the amount of data transferred to a cloud and delivers services to many devices with powerful wireless connections. For example, traffic control can be automated by collecting data from cameras and sensors installed at a side of road in real-time. Sensors detect approaching objects including pedestrians and vehicles and measure the distance from/to or the speed of the objects. They send a signal to a smart traffic light to provide appropriate instructions to vehicles, based on the collected information. Although the areas covered by the edge layer are different, they could provide low network latency, sensor position recognition, wide geographical distribution and mobile service.

### 3.2. Communication Control by Power Consumption

Low performance IoT devices have low calculation and networking capability. Therefore, selected representative sensors are used as a small unit of proxy server to save power. In other words, m-proxy servers from selected sensors become a small unit of proxy server. Through this setting, a proxy server can manage devices.

In sensor networks, the layer-based routing protocol is LEACH-C, which improves LEACH. This method selects m-proxy servers according to energy consumption. However, all the sensor devices have to communicate with the proxy server in every round, and there is overhead for additional energy consumption and location processing.

Therefore, this paper proposes a more effective information collection method. We estimate power consumption by m-proxy servers and normal devices in an early round and expect the remaining power level for the next round to remove unnecessary communications from LEACH-C.

**Figure 9:**
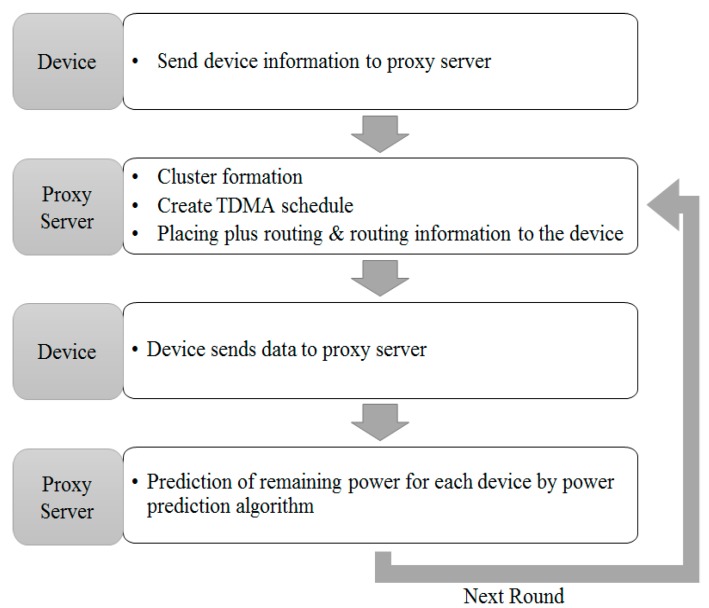
Model of Energy Level Estimation.

An equation may provide an example. Figure 9 indicates the structure of a model to estimate device’s power consumption. In the first round, a sensor device transfers its current position and the remaining power level to the proxy server. The proxy server configures the best cluster with information received, creates a Time Division Multiple Access (TDMA) schedule and broadcasts it to all devices. Then, the device transmits its own sensing data to the proxy server through the cluster structure. This is the end of one round. From the next round, the proxy server configures clusters through power consumption estimation algorithm, without making unnecessary communications to collect device information:

*E_Tx_(l, d) = E_Tx-elec_ (l) + E_Tx-amp_ (l, d)*, (1)

where: *E_Tx-elec_ (l)*: Power consumption to transmit a message with Size 1, *E_Tx-amp_ (l)*: Increased power consumption to send a message with Size 1 for ‘D’ distance.

Equation (1) is the power consumption estimation algorithm for a device. It calculates the power consumption to send a 1 bit message over a distance ‘d’. At the setting stage of two early rounds, the proxy server is set to receive the expected power consumption from all devices. From the next round, the proxy server is set to not receive current power consumption from all devices. The power consumption could be estimated. Therefore, only at the setting stage of the two most early rounds, is the energy level of all devices received. In the next round, the current energy level is not transmitted from all devices. However, the remaining energy level could be estimated from the early rounds. Therefore, power consumption of a device after a round can be calculated. With this power consumption, we can estimate the average power consumption of a small unit of proxy server and a normal sensor. In the next round, the current energy level can be estimated by subtracting estimated power consumption from the energy level of the previous round.

Continual use of estimated power consumptions may cause an error. To prevent errors from being accumulated, regular synchronization is necessary.

**Figure 10:**
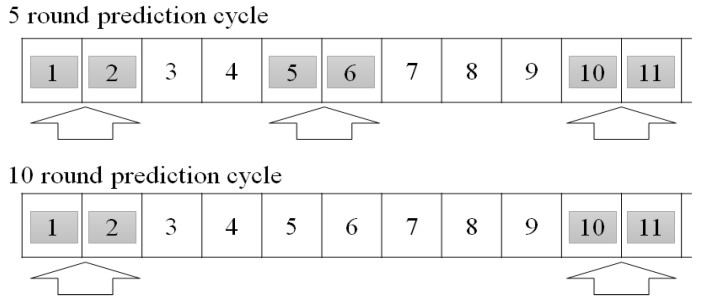
Round-by-Prediction Cycle.

For the cycle of five rounds, as shown on the top of Figure 10, an energy level is transmitted from all devices, and the average power consumption of a small unit of proxy server and a normal sensor device are calculated at the first and the second round. The calculated power consumption is used to estimate an energy level without making communication during the third and the fourth rounds. Then, in the fifth and the sixth round, an energy level is transmitted for synchronization, and the power consumption is calculated. With the result, estimation is conducted in rounds 7 and 8. This could save energy accounting for power consumption for six rounds in the complete cycle of 10 rounds.

On the other hand, for the cycle of 10 rounds, as shown in the bottom of Figure 10, an energy level is transmitted from all devices, and the average power consumption of a small unit of proxy server and a normal sensor device are calculated at the first and the second round. The calculated power consumption is used to estimate an energy level, without making communication at rounds 3, 4, 5, 6, 7, 8 and 9. Then, an energy level is transmitted for synchronization at rounds 10 and 11 and the power consumption is calculated. With the result, estimation is conducted in rounds 12, 13, 14, 15, 16, 17, 18 and 19. In other words, this could save energy accounting for power consumption for eight rounds in the complete cycle of 10 rounds.

However, it cannot be said that the cycle of 10 rounds is more effective than the cycle of five rounds in terms of energy consumption because the cycle of 10 rounds could have bigger error tolerance than the cycle of five rounds. As the overall network life-time is more important than communication of an individual device in a sensor network, it is important to find the optimized estimation cycle according to the errors. According to the experiments, the cycle of eight rounds shows the best performance.

### 3.3. Communication Control by Frequency of Use

When data is transferred from a sensor to the proxy server, data information is processed in the fog area. The processed data is transmitted to the cloud server and printed to a device or a user by an order created by the cloud server or the proxy server. Data processed in fog computing is directly transmitted to cloud communication. However, such communication with the cloud server wastes cloud server capacity and power consumption due to unnecessary data communication.

**Figure 11:**
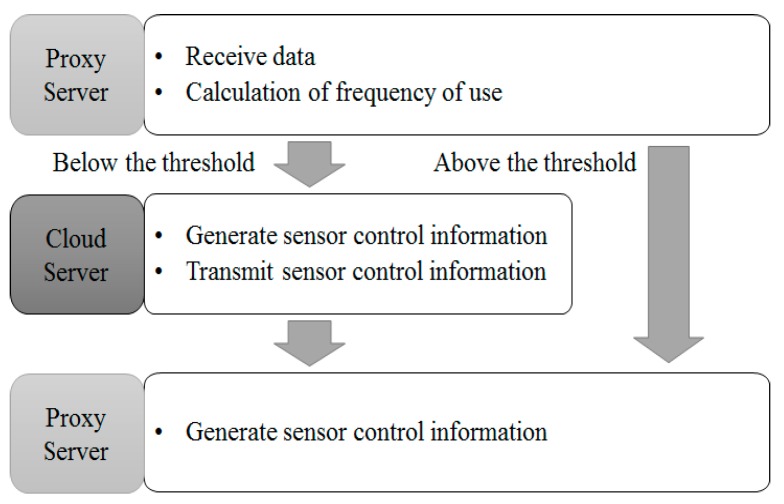
Structure of Usage-Based Communication Control.

To solve the issue, this paper proposes a structure, on the basis of frequency of use in data information processing process, as indicated in Figure 11. First, data is received. Next, we measure frequency of data use. If the measured frequency of use meets the standard, we skip communication with the cloud server, create control information and the proxy server and transmit it to sensors or devices. If the measured frequency of use does not meet the standard, we communicate with the cloud server and receive information from the cloud server, creating control information accordingly and sending sensor control information to the proxy server. If the standard is drawn from calculations, information satisfying the standard is directly transmitted to the proxy server, without communication with the cloud server. Therefore, the number of communications with the cloud server and power consumption can be reduced. In addition, as communications with the cloud server happen only when data does not satisfy the standard and has a problem, unnecessary communication does not occur. As a result, the amount of data (load) received by the cloud server is reduced.

### 3.4. Communication Control by Access Authority

A system adopting user attribute-based encryption sends data to a server via an access structure after encryption. A user who wants to share and use encrypted data of a service user requests decryption authority from a proxy server. A service user decides whether a person who requests decryption authority is a legitimate user and creates an attribute delegated key and sends it to a proxy server. In this system, a proxy server should have an attribute delegation list and an attribute revocation list. A proxy server checks the attribute delegation list and re-encrypts data using an attribute delegated key before providing it to a user.

**Figure 12:**
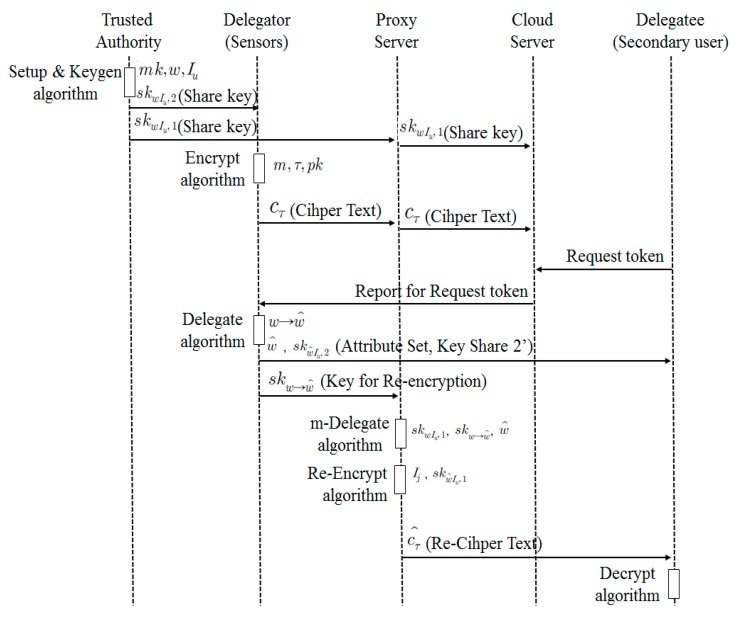
Flow of Communication Control for Access Authority.

As shown in Figure 12, system components include a trusted authority, sensors, a proxy server, a cloud server and secondary user. Sensors generate data. A sensor can be a delegator that can delegate an authority. A secondary user is a user who can utilize information collected from a sensor. However, they need an authority of decryption before using data. They can be a delegatee. A proxy sever collects and processes data transmitted from a sensor. Therefore, it should have sufficient memory and processing capability. In addition, it can re-encrypt data or process it with collected data, depending on circumstance. A cloud server processes information collected from a sensor. Furthermore, it collects and analyzes data received from a proxy server. It makes a decision using the results of analysis. It should learn information about decision making in advance to understand the personal data and effectively process it. It manages attribute delegation and revocation and monitors data.

There are roughly six service scenarios using attribute-based encryption technology that allow delegation and revocation of an attribute.

- A Trusted Authority (TA) defines system parameters using Setup (*k*) algorithm and creates the public key (*pk*) and the master key(*mk*). In addition, it creates two private key shares *sk_wIu,_*_1_ and *sk_wIu,_*_2_ relating to Attribute *w* and Public Key *I_u_* and sends *sk_wIu,_*_1_ to a proxy server and *sk_wIu,_*_2_ to a sensor or a data owner, using KeyGen (*mk*, *w*, *I_u_*) algorithm.
- A user transmits Ciphertext *c_τ_* that encrypts Data *m* using Encrypt (*m*, *τ*, *pk*) algorithm. In this context, data refers to sensing data.
- A secondary user requests a decryption token (attribute set and ciphertext) to a proxy server to access Data *m* of the data owner.
- The data owner defines *w′* based on his/her own attribute set *w* to delegate decryption authority for Ciphertext *c_τ_* to a secondary user. The data owner creates the private key share for a secondary user *sk_wIu,_*_2_ and the proxy server key to delegate an attribute *sk_w→w′_* using his/her own private key share *sk_wIu,_*_2_ and the public key for a secondary user *I_j_*, and send them a secondary user (*w’*, *sk_w’Iu,_*_2_) and the proxy server (*sk_w→w′_*).
- A proxy server creates its own private key share *sk_wIu,_*_1_, proxy key *sk_w→w′_* and the private key share for a secondary user *sk_w’Iu,_*_1_ using the attribute set *w′* defined by a patient. It re-encrypts the ciphertext *c_τ_* with the public key for a secondary user *I_j_*, *sk_w’Iu,_*_1_ and sends the re-encrypted ciphertext *c_τ′_* to a secondary user.
- A secondary user obtains the data *m* by decoding the ciphertext *c_τ′_* using the key received from the proxy server and the data owner *sk_w’Iu,_*_2_.

## 4. Experiment and Analysis

### 4.1. Performance of Proposed Fog Computing

The fog network was tested by the iFogSim Toolkit. iFogSim provides functions to simulate all network nodes and print the simulation results. For comparison of all fog computing data, the cloud computing and the computing system adopting fog layer were compared. The number of sensors was total 50, 100, 150 or 200. A proxy server was configured, combining four segments. The CPU of each sensor was 1.0 GHz. The size of data to be transferred was set to 20 KB. The average transmission time between network devices was 5 ms.

**Figure 13:**
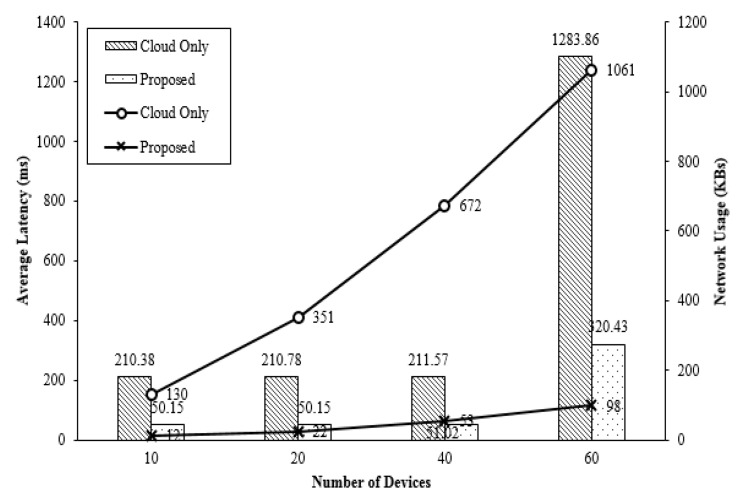
Average Latency and Network Usage Comparison.

Compared to the event that uses cloud computing only, it is shown that the system using the fog layer has reduced waiting time and network usage, which is caused by communication control between a fog computer and sensors. Figure 13 demonstrates comparison of energy consumption at an end point, an edge device and a data center between cases when only cloud is used or fog calculation is used. Figure 13 also indicates power consumption of fog computing architecture that uses a cloud. For the fog layer, power was mainly consumed at sensors that perform most processing works while a data center or a cloud consumed most energy in the cloud computing.

### 4.2. Sensor Network Performance by Communication Control

**Figure 14:**
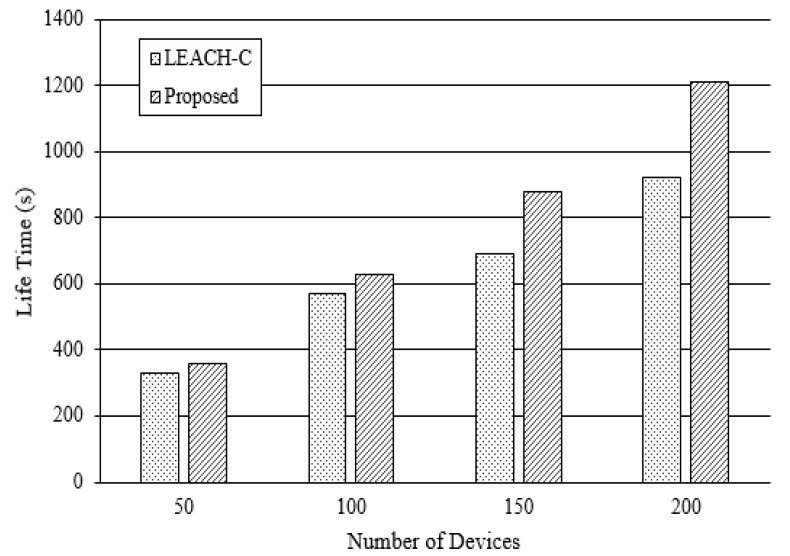
Life Time Comparison of Devices.

To measure the performance of the sensor network, it was tested, separate from the overall system. NS-3 network simulator was used to test the sensor network. For comparison, the LEACH-C module, a sensor network hierarchical management method, was used. The number of sensors was increased to 50, 100, 150 and 200. Transmission speed was set to 1 Mbps on the sensor field (100 m × 100 m area). Sensors were fixed for an effective test. Figure 14 provides the life time by the number of devices. Life time was increased by 9.43%, 12.27%, 21.17% and 24.41%, respectively. This result indicates that the proposed algorithm shows better performance when there are more devices.

### 4.3. Security Analysis

This paper verifies whether the proposed method is safe, assuming two possible events that could happen during attribute revocation process. The validation items are analyzed for the events when a revoker presents a random value or a value to renew an attribute value is exposed.

First, there could be two different cases when a revoker presents a random value. A revoker can present a random value or a modified value during the process of attribute value renewal for continuous renewal. In this case, an attacker could not know the random value. Therefore, it is impossible for an attacker to disguise as a non-revoker by modifying the random value. In other words, it is impossible to find a correct value during the renewal process with a value modified by an attacker. This is also true in the case when an attacker randomly changes a certain value by multiplication or division.

Second, it is assumed that a value to renew an attribute value is exposed when a message for attribute value renewal is being received or during the renewal process of an attribute value that is not revoked. When a renewal value is exposed, an attacker may attempt to renew an attribute value that was previously revoked by him or her. However, it is obvious that the value calculated in this process differs from the value obtained from a correct renewal process. In addition, an attacker does not know a default value. Therefore, it is impossible for an attacker to renew a renewal value even if the value is exposed during the process.

## 5. Conclusions

Cloud computing provides data content and control information to devices based on centralized data access and delivery structure. With cloud computing, a huge amount of data is transmitted and processed. There are also problems such as delays in service response time. In addition, as the Internet of Things develops, the application fields of sensor networks are further expanded, and demands for real time processing, transmission, and security of information controlling object Internet devices are increasing. Fog computing has emerged as a way to effectively address this problem. Fog computing is also referred to as edge computing, and includes a function of processing information collected from a device before sending it to the cloud to reduce the amount of transmission or performing itself without receiving device control information from the cloud. Thus, the data transmission delay in the device is reduced and the device can be controlled in real time.

With the recent increases in the number of devices, the power consumption in the communication between the sensors in the fog computing environment is increased. Various studies have been conducted to improve the performance in various scenarios and service delays in fog computing, but research on effective power reduction is insufficient.

This paper presents a power consumption problem in the fog computing environment and suggests an effective method for power reduction. We also propose a method for securely managing the data generated by each sensor.

In order to solve the power consumption problem, a proxy server is placed between the cloud server and the sensor, and the proxy server processes the data according to the frequency of use of the data, thereby reducing the load of the entire communication. In addition, effective power reduction is achieved between the proxy server and the sensor by hierarchically managing it according to power consumption. Experimental results show that the total network latency and network usage are reduced. The system encrypts data based on the user’s attributes so that only authorized users can use the information. In addition, it performs efficient data management by adding attributes and revocation. As a result of verifying the problems that may occur in the process of withdrawing the attribute value of the user through the security evaluation, it can be seen that it is safe because the property value cannot be falsified or the attribute cannot be updated at random.

Future research needs to be applied to a sensor network that can move freely in the proposed method through simulation. And intelligent data transmission methods based on information- centric networks using context-aware technology.

## Figures and Tables

**Table 1:** CISCO’s Internet of Things Reference Model.

Layer	Name	Detailed Role
1	Physical Devices & Controllers	The “Things” in IoT
2	Connectivity	Communication, Processing Units
3	Fog Computing	Data Element Analysis, Transformation
4	Data Accumulation	Storage
5	Data Abstraction	Aggregation, access
6	Application	Reporting, Analytics, Control
7	Collaboration & Processes	Involving People, Business Processes
